# Supplementary material for: Spatial Distribution and Retention in Loblolly Pine Seedlings of Exogenous dsRNAs Applied through Roots
Source: Int J Mol Sci. 2022 Aug 15;23(16):9167. doi: 10.3390/ijms23169167 (PMC9409306; doi:10.3390/ijms23169167)
Supplement: Supplementary file 1 [file ijms-23-09167-s001.zip › ijms-1856673-supplementary.pdf]

International Journal of *Molecular Sciences*

MDPI

Supplementary Materials

Table S1. Recovery of dsRNA from plant material via amplicon presence on gel images. Presence scores listed in numerator and total tissue samples in denominator. (**a**) Combined dsRNA recovery between both treatments (dsSHI and dsGFP) (**b**) recovery of dsSHI replicates combined (**c**) recovery of dsGFP replicated combined (**d**) recovery of dsSHI by replicate (**e**) recovery of dsGFP by replicate.

(a)	24 h	72 h	120 h	168 h	Combined
**Root**	10/18	7/18	0/18	1/18	18/72
**Stem**	12/18	9/18	3/18	1/18	25/72
**Crown**	9/18	6/18	3/18	2/18	20/72
**Needle**	10/18	3/18	3/18	3/18	19/72
**Meristem**	11/18	3/18	3/18	2/18	19/72
**Combined**	52/90	28/90	12/90	9/90	101/360

(b)	24 h	72 h	120 h	168 h	Combined
**Root**	5/9	4/9	0	0	9/36
**Stem**	5/9	6/9	0	0	11/36
**Crown**	3/9	3/9	0	0	6/36
**Needle**	3/9	0	0	0	3/36
**Meristem**	4/9	0	0	0	4/36
**Combined**	20/45	13/45	0/45	0/45	33/180

(c)	24 h	72 h	120 h	168 h	Combined
**Root**	5/9	3/9	0/9	1/9	9/36
**Stem**	7/9	3/9	3/9	1/9	14/36
**Crown**	6/9	3/9	3/9	2/9	14/36
**Needle**	7/9	3/9	3/9	3/9	16/36
**Meristem**	7/9	3/9	3/9	2/9	15/36
**Combined**	32/45	15/45	12/45	9/45	68/180

(d)	1 d			3 d			5 d			7 d		
**Rep**	1	2	3	1	2	3	1	2	3	1	2	3
**Root**	3/3	2/3	0	2/3	2/3	0	0	0	0	0	0	0
**Stem**	3/3	2/3	0	3/3	3/3	0	0	0	0	0	0	0
**Crown**	2/3	1/3	0	1/3	2/3	0	0	0	0	0	0	0
**Needle**	1/3	2/3	0	0	0	0	0	0	0	0	0	0
**Meristem**	2/3	2/3	0	0	0	0	0	0	0	0	0	0

(e)	1 d			3 d			5 d			7 d		
**Rep**	1	2	3	1	2	3	1	2	3	1	2	3
**Root**	3/3	0	2/3	3/3	0	0	0	0	0	1/3	0	0
**Stem**	3/3	1/3	3/3	3/3	0	0	3/3	0	0	1/3	0	0
**Crown**	2/3	1/3	3/3	3/3	0	0	3/3	0	0	2/3	0	0
**Needle**	3/3	1/3	3/3	3/3	0	0	3/3	0	0	3/3	0	0
**Meristem**	3/3	1/3	3/3	3/3	0	0	3/3	0	0	2/3	0	0

Table S2. Logistic regression models of dsRNA recovery in each tissue type. The reference category is unsuccessful recovery of dsRNA from treated tissue, so odds ratios represent the effect a given character has on the likelihood that dsRNA would be successfully recovered from a treated tissue type. Dashes are present for both dsGFP and Replicate 1 as each serve as the reference category for the categorical variables to which they belong. Root collar diameter is abbreviated RCD. Numerals under Replicate correspond to each of the three replications of the study. Odds ratios calculated by raising e^x where x equaled model coefficients; 95% confidence interval given for individual odds ratios; bolded p-values represent significance at $p < 0.05$.

	Root			Stem			Crown			Needle			Meristem		
**Characteristic**	**OR**	**95% CI**	**p-value**	**OR**	**95% CI**	**p-value**	**OR**	**95% CI**	**p-value**	**OR**	**95% CI**	**p-value**	**OR**	**95% CI**	**p-value**
**Time (h)**	0.96	0.93, 0.98	**<0.001**	0.95	0.92, 0.98	**<0.001**	0.98	0.96, 0.99	**0.007**	0.97	0.95, 0.99	**0.005**	0.96	0.94, 0.98	**0.001**
**RCD (mm)**	0.32	0.04, 1.81	0.25	0.82	0.15, 3.66	0.80	0.67	0.16, 2.46	0.57	0.64	0.12, 3.05	0.58	0.79	0.15, 3.59	0.76
**Height (cm)**	1.16	0.95, 1.46	0.17	1.00	0.82, 1.23	>0.99	1.04	0.88, 1.22	0.67	1.00	0.82, 1.20	0.99	0.96	0.79, 1.16	0.68
**RNA (ng/uL)**	0.99	0.97, 1.01	0.44	1.00	0.99, 1.02	0.75	0.99	0.98, 1.00	0.22	0.99	0.98, 1.01	0.33	0.99	0.98, 1.00	0.29
**Replicate**															
1	—	—		—	—		—	—		—	—		—	—	
2	0.07	0.00, 0.57	**0.025**	0.02	0.00, 0.22	**0.007**	0.16	0.02, 1.12	0.081	0.03	0.00, 0.27	**0.007**	0.03	0.00, 0.27	**0.007**
3	0.03	0.00, 0.32	**0.009**	0.01	0.00, 0.08	**0.001**	0.13	0.01, 1.08	0.074	0.04	0.00, 0.57	**0.035**	0.04	0.00, 0.78	0.054
**Treatment**															
dsSHI	—	—		—	—		—	—		—	—		—	—	
dsGFP	0.90	0.18, 4.43	0.90	0.41	0.08, 1.84	0.26	0.18	0.04, 0.70	**0.02**	0.02	0.00, 0.13	**<0.001**	0.03	0.00, 0.19	**0.002**
**Efron's Pseudo R^2**		0.50			0.53			0.37			0.54			0.56	

OR = Odds Ratio, CI = Confidence Interval

Table S3. Primer sequences for target genes for PCR amplification and dsRNA synthesis. Promoter sequence of T7 RNA Polymerase in bold. Amplicon size corresponds to final dsRNA length.

Gene	Type		Primer Sequence 5′3′	Amplicon
shi	qPCR	**F**	TACTTCTTTTCGCGCTCCTC	109
		**R**	GCATCCATAATCTGGGCATC	
	PCR/ dsRNA	**F**	**TAATACGACTCACTATAGGG**AGTTCGCCGTTGATGAAATC	370
		**R**	**TAATACGACTCACTATAGGG**TCGAGCAGGGCTTTATGTCT	
gfp	qPCR	**F**	GCTGACCCTGAAGTTCATCT	99
		**R**	TAGCGGCTGAAGCACTG	
	PCR/ dsRNA	**F**	**TAATACGACTCACTATAGGG**CGATGCCACCTACGGCAA	248
		**R**	**TAATACGACTCACTATAGGG**TGTCGCCCTCGAACTTCA	
18s	qPCR	**F**	CTGCGCGTTGTATCGAATTA	85
		**R**	ACGGTCGCAAGACTGAAACT	
	PCR	**F**	**TAATACGACTCACTATAGGG**CCAGGGTATCTGATCCTGTTTG	383
		**R**	**TAATACGACTCACTATAGGG**CAATATCGCGTGGGTGAAGA	

Table S4. dsRNA concentrations and total volume used per seedling to attain 250 ng treatments.

	dsRNA	Concentration	Volume Delivered
Replicate 1	dsSHI	114.0 ng/μL	2.19 μL
	dsGFP	94.8 ng/μL	2.64 μL
Replicate 2	dsSHI	102.5 ng/μL	2.44 μL
	dsGFP	102.1 ng/μL	2.45 μL
Replicate 3	dsSHI	76.8 ng/μL	3.26 μL
	dsGFP	109.1 ng/μL	2.29 μL

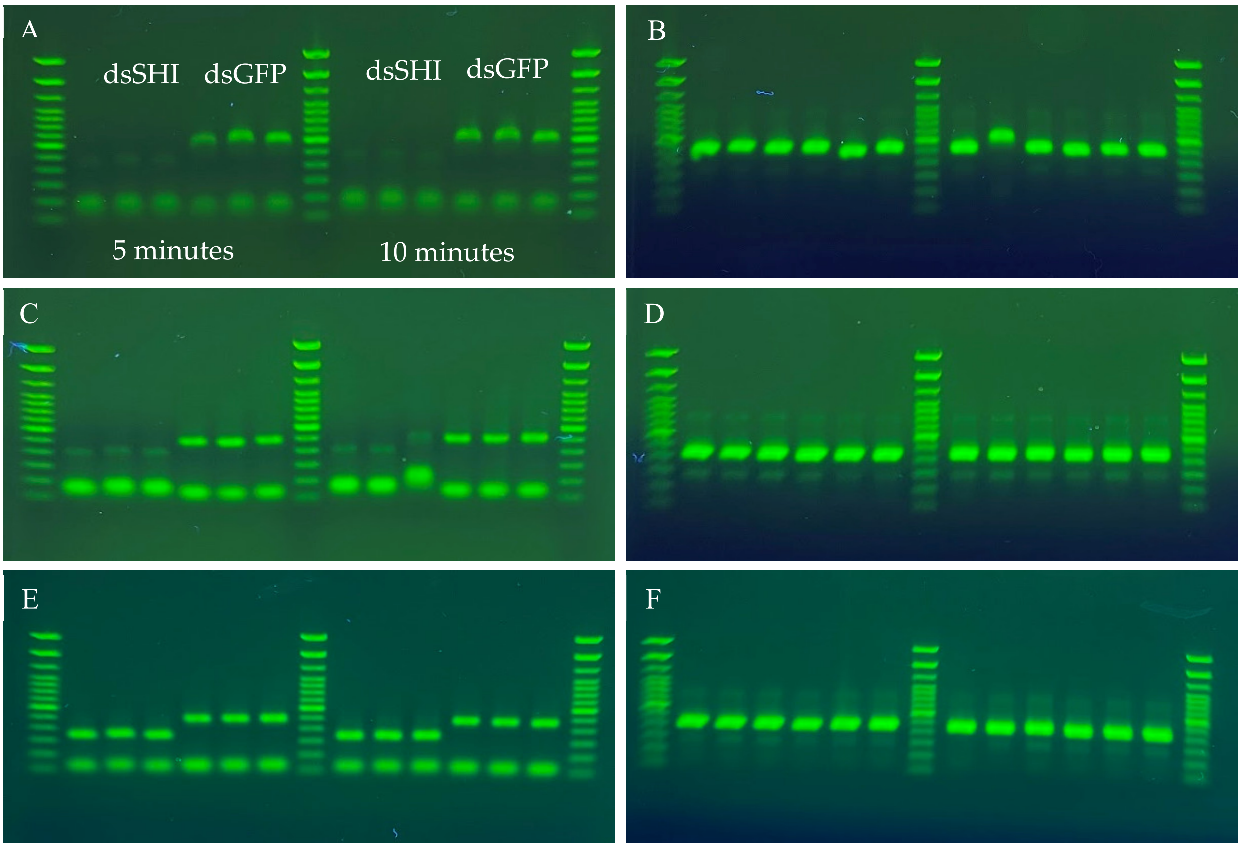

Figure S1. Gel images of PCR products from cDNA incubation trial. cDNA incubation temperatures consisted of 65C, 75C, and 90C with incubation time intervals of both 5 and 10 minutes. cDNA was synthesized from a mixture of 3.5 uL of template RNA consisting of 1 uL of dsRNA (50 ng) and 2.5 uL of loblolly RNA (500 ng). Following cDNA synthesis PCR was conducted on each sample using primers for both an endogenous control (18s) and the target dsRNA. PCR products shown in (**A** & **B**) were generated with a cDNA annealing temperate of 65C, (**C** & **D**) 75C, and (**E** & **F**) 90C. For all gel images, the left portion was incubated during cDNA synthesis for 5 minutes and the right portion 10 minutes, as labelled in (**A**). For images **A**, **C**, and **E**, each subset of samples are further divided with three samples of dsSHI (on the left) and three of dsGFP (on the right), as demonstrated in (**A**). Images **B**, **D**, and **F** contain samples of the endogenous control (18s) amplification.

Table S5. Specific sample information for Sanger sequencing products.

Amplicon Name	Sequenced sample
Treatment-SHI	Stock purified PCR product used as template to make dsSHI
Recovered-SHI	PCR product amplified using dsSHI primers on root tissue treated with dsSHI (Replicate 1, dsSHI seedling 1, root tissue, 1 day exposure)
Control-18s-SHI	PCR product amplified using 18s primers on root tissue treated with dsSHI (Replicate 1, dsSHI seedling 1, root tissue, 1 day exposure)
Treatment-GFP	Stock purified PCR product used as template to make dsGFP
Recovered-GFP	PCR product amplified using dsGFP primers on root tissue treated with dsGFP (Replicate 1, dsGFP seedling 1, root tissue, 1 day exposure)
Control-18s-GFP	PCR product amplified using 18s primers on root tissue treated with dsGFP (Replicate 1, dsGFP seedling 1, root tissue, 1 day exposure)
